# RNA polymerase II depletion promotes transcription of alternative mRNA species

**DOI:** 10.1186/s12867-016-0074-8

**Published:** 2016-08-30

**Authors:** Lijian Yu, Mayuri Rege, Craig L. Peterson, Michael R. Volkert

**Affiliations:** 1 Microbiological and Physiological Systems, University of Massachusetts Medical School, 55 Lake Avenue North, Worcester, MA 01655 USA; 2 Program in Molecular Medicine, University of Massachusetts Medical School, 373 Plantation Street, Worcester, MA 01605 USA

**Keywords:** Transcription, RNA polymerase depletion, Transcriptional stress, Altered polyadenylation preference, mRNA induction

## Abstract

**Background:**

Cells respond to numerous internal and external stresses, such as heat, cold, oxidative stress, DNA damage, and osmotic pressure changes. In most cases, the primary response to stress is transcriptional induction of genes that assist the cells in tolerating the stress and facilitate the repair of the cellular damage. However, when the transcription machinery itself is stressed, responding by such standard mechanisms may not be possible.

**Results:**

In this study, we demonstrate that depletion or inactivation of RNA polymerase II (RNAPII) changes the preferred polyadenylation site usage for several transcripts, and leads to increased transcription of a specific subset of genes. Surprisingly, depletion of RNA polymerase I (RNAPI) also promotes altered polyadenylation site usage, while depletion of RNA polymerase III (RNAPIII) does not appear to have an impact.

**Conclusions:**

Our results demonstrate that stressing the transcription machinery by depleting either RNAPI or RNAPII leads to a novel transcriptional response that results in induction of specific mRNAs and altered polyadenylation of many of the induced transcripts.

**Electronic supplementary material:**

The online version of this article (doi:10.1186/s12867-016-0074-8) contains supplementary material, which is available to authorized users.

## Background

UV damage causes lesions in the genome that interfere with cellular DNA metabolism and lead to cell death. UV damage also induces a DNA repair response that triggers a cascade of events, including transcriptional activation of a large number of DNA damage response genes that facilitate DNA repair, and trigger cell cycle arrest until DNA repair is complete [1, 2]. In contrast, the presence of UV lesions in DNA also causes RNA polymerase arrest, blocking elongation and thus, antagonizes genome-wide transcription [3–5]. The arrest of RNAP II initiates a DNA repair response known as transcription coupled repair (TCR). RNA polymerase and associated proteins serve as the DNA damage recognition factor for TCR, recruiting nucleotide excision repair proteins to preferentially repair DNA damage located in the template strand of actively transcribing DNA [4]. The arrest of multiple RNA polymerase complexes at DNA damage sites throughout the genome also reduces the number of functionally active and free, RNA polymerase molecules available.

The *RPB2* gene locus has served as an ideal system to investigate effects of UV damage in the cell [3, 6–8]. Given the thorough investigation of transcription of this gene after UV treatment, our recent work on recovery after UV damage at *RPB2* revealed an unexpected transcriptional response. Prior to UV treatment, the *RPB2* mRNA terminated at a polyA site that is 58 nt downstream from the stop codon. Shortly after UV treatment, a distal polyA site 345 nt downstream of the stop codon is preferentially used, resulting in a longer transcript. Moreover, the abundance of this *RPB2* long form increased markedly over the first 60 min after UV. This increase was not due to stabilization of the longer *RPB2* transcript, as the half-lives of both the short and long form of *RPB2* transcripts were comparable. Thus, UV treatment induced transcription of the *RPB2* gene and the mRNA that was produced preferentially utilized a distal polyA site [9]. Given that UV treatment generally inhibits transcription genome-wide until the damage is repaired, this serendipitous observation was quite remarkable.

In this study we extend this work to test whether production of the *RPB2* long form is a general hallmark of transcriptional stress. We also examine if other genes show a similar response, and if depletion or inactivation of other RNA polymerases serves as an inducer of the response. We find that inactivation of RNAPII or nuclear depletion of either RNAPI or RNAPII triggers transcriptional changes similar to the changes seen after UV treatment. Thus it appears that treatments that reduce the level of free or active transcription complexes cause a type of transcriptional stress that triggers induction of specific genes and modulation of polyadenylation (polyA) site usage.

## Results

### Depletion of RNA polymerase II induces the long form of *RPB2* mRNA

**Fig. 1:**
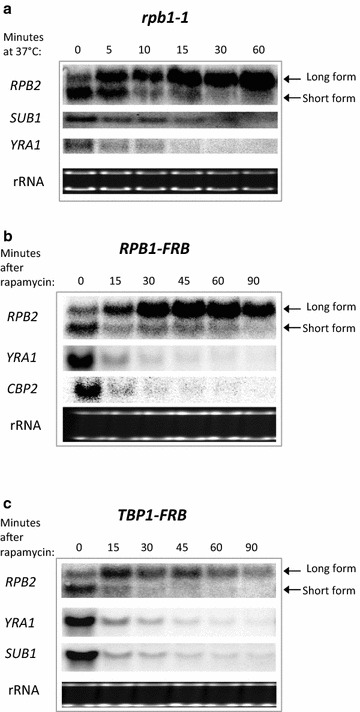
Polymerase stress increases transcription of the long *RPB2* mRNA. **a** Northern analysis [29] of mRNA levels when shifting the *rpb1*-*1* mutant to the non-permissive temperature of 37 °C. Northern blot analysis of mRNA levels in the **b** RNAPII anchor-away cells (*RPB1*-*FRB*; MVY859) and **c** Tbp1 anchor-away cells (*TBP1*-*FRB*; MVY868) after addition of rapamycin to deplete *FRB* tagged proteins from the nucleus. The long and short isoform of the *RPB2* mRNA are indicated

UV damage has both positive and negative effects on transcription. It triggers a UV induced DNA-damage response that stimulates transcription of genes required for DNA repair and cellular recovery while the presence of lesions in the template DNA stalls transcription throughout the genome [3–5]. Our previous study demonstrated several additional changes in transcription after UV treatment. Specifically, the polyA site preference at the *RPB2* gene shifts from production of a 4010 nt mRNA to a longer 4297 nt mRNA, and transcription of the long form is induced dramatically [9]. One possibility is that this transcriptional change is due to a direct response to UV damage. Alternatively, induction of the long form of *RPB2* may be due to the general inhibition of transcription that results from UV treatment [3–5]. As an initial test of the latter possibility, the *rpb1*-*1* temperature sensitive allele of the gene encoding the largest subunit of RNAPII (Rpb1/Rpo21) was used to rapidly inactivate RNAPII at 37 °C [10], and the abundance of *RPB2* mRNA species was analyzed by Northern analysis. When transcription is inhibited by incubating the *rpb1*-*1* mutant at 37 °C, changes in *RPB2* expression are observed that are similar to those seen after UV treatment. The expression of the 4297 nt long *RPB2* mRNA increases after Pol II inactivation, while levels of the shorter 4010 nt *RPB2* mRNA decrease (Fig. 1a). As would be expected from a genome-wide inactivation of the transcriptional machinery, levels of other control mRNAs, such as *SUB1* and *YRA1* decline. This indicates that the inactivation of RNAPII results in the expected inhibition of general transcription, but with the striking exception that transcription of the long *RPB2* mRNA was not repressed at the restrictive temperature but rather appears to be induced.

To test if simply depleting RNAPII from the nucleus is sufficient to induce transcription of the long form of *RPB2*, we employed the anchor-away technique, which is a rapamycin-inducible system that rapidly depletes nuclear proteins to the cytoplasm [11]. A previous study used the anchor away method to demonstrate that RNAPII can be depleted from genes, and RNAPII transcription is reduced within 15 min of rapamycin addition. Likewise, we find that nuclear depletion of the largest subunit of RNAPII, Rpb1, leads to a rapid reduction in *CBP2* and *YRA1* mRNAs (*RPB1*-*FRB* strain, Fig. 1b). A similar reduction in transcription is also seen for the short *RPB2* mRNA (Fig. 1b). In contrast, the long *RPB2* mRNA is not inhibited, but instead its synthesis increases over time after depleting RNAPII. Thus, it appears that depletion of actively transcribing RNAP II complexes from the nucleus triggers the observed changes in polyA site preference and induction of *RPB2* transcription.

As an independent means to deplete RNAPII from target genes, the anchor away strategy was used to deplete the general RNAPII transcription factor, TATA binding protein 1 (*Tbp1*). Tbp1 depletion leads to the expected decrease in expression of several genes, including the short form of *RPB2* (*TBP1*-*FRB* strain, Fig. 1c). In contrast, Tbp1 depletion led to an increase in expression of the long *RPB2* mRNA, although expression of the long form of *RPB2* slowly declined with time. Thus, like the case for depletion or inactivation of RNAPII, depletion of Tbp1 results in an induction of the long form of *RPB2*.

### Induction of the long form of *RPB2* is not due to increased RNAPII occupancy

**Fig. 2:**
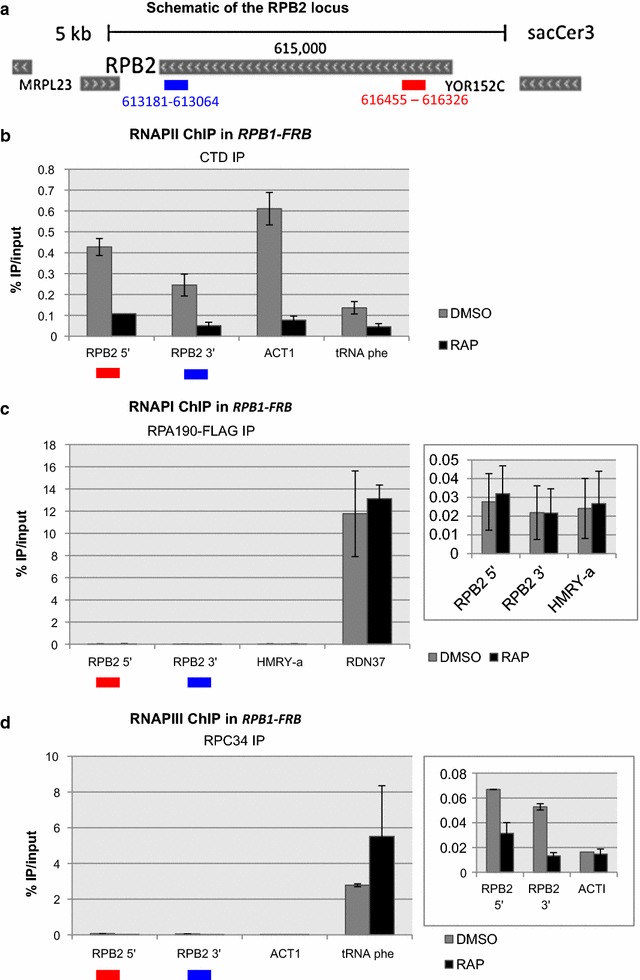
Chromatin Immunoprecipitation of RNA polymerase subunits at the *RPB2* locus after RNAPII depletion. **a** The schematic indicates locations of the primers used for chromatin immunoprecipitation at the 5′ and 3′ end of the RPB2 locus. IP was performed using the **b** anti-CTD antibody for RNAPII. **c** anti-FLAG antibody for the Rpa190-FLAG subunit of RNAPI and **d** anti-Rpc34. for RNAPIII. *RDN37* and tRNA phe are known targets of RNAPI and RNAPIII respectively and thus were used as positive controls

The induction of the long form of *RPB2* mRNA following inactivation or depletion of RNAPII raises the possibility that the residual RNAPII is preferentially retained, or accumulates, at the *RPB2* locus. To test this possibility, chromatin immunoprecipitation (ChIP) was used to measure RNAPII levels at the *RPB2* locus in *RPB1*-*FRB* cells treated with either DMSO or rapamycin for one hour. Contrary to our expectation, rapamycin addition led to a rapid decrease in RNAPII occupancy throughout the *RPB2* coding region, similar to other genes (Fig. 2b; Additional file 1: Figure S1). Thus, either the residual RNAPII remaining at the *RPB2* locus is actively engaged in transcription, or other RNA polymerases substitute for RNAPII when it is depleted or inactivated. To determine if other polymerases induce this response and to test whether other RNAPs are required for the increased expression of the large *RPB2* transcript, we examined the effects of depleting or inactivating RNAPI or RNAPIII.

### Depletion of RNAPI promotes expression of the long form of *RBP2*

**Fig. 3:**
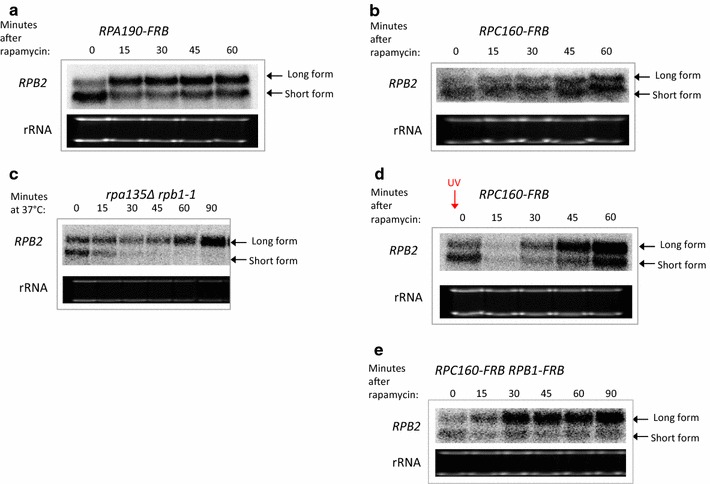
Depletion of RNAPI but not RNAPIII induces the long *RPB2* mRNA. **a** Nothern analysis [29] of the *RPB2* mRNA isoforms after **a** RNAPI anchor-away (*RPA190*-FRB; MVY860), **b** RNAPIII anchor-away (*RPC160*-*FRB*; MVY862). **c** Northern analysis of the *RPB2* mRNA isoforms after heat inactivation of RNAPII in the *rpa135Δ rpb1*-*1* (MVY851). **d** Northern analysis of the *RPB2* mRNA isoforms after RNAPIII anchor-away (*RPC160*-*FRB*; MVY862), except that cells are UV irradiated (70 J/m^2^) before adding rapamycin. **e** Northern analysis of the *RPB2* mRNA isoforms after depletion of RNAPII and RNAPIII simultaneously (*RPA190*-*FRB RPB1*-*FRB;* MVY872)

The anchor away strategy was used to test if depletion of either RNAPI or RNAPIII might also trigger the change in *RPB2* transcription. Surprisingly, depletion of the Rpa190 subunit of RNAPI led to an increase in the level of the long *RPB2* mRNA, while the short form of *RPB2* was decreased only slightly (Fig. 3a). In contrast, depletion of the Rpc160 subunit of RNAPIII had no impact on either the short or long forms of *RPB2* (Fig. 3b). Thus, depletion of either RNAPI or RNAPII causes increased expression of the long form of *RPB2,* whereas depletion of RNAPIII has no effect.

Since RNAPI and RNAPIII share multiple subunits with RNAPII, we tested the formal possibility that RNAPI or RNAPIII might substitute for RNAPII at the *RPB2* gene after RNAPII depletion. However, ChIP analysis of the Rpa190 subunit of RNAPI and the Rpc34 subunit of RNAPIII did not detect an increase in their levels at the *RPB2* locus following depletion of RNAPII (Fig. 2c, d). Several functional studies were also used to probe whether RNAPI or RNAPIII might play a more direct role in *RPB2* transcription under conditions where RNAPII is depleted or inactivated. To test if RNAPI plays a role in gene expression after transcriptional stress, we used a strain that carries a deletion of the *RPA135* gene (*rpa135∆*) that encodes an essential subunit of RNAPI. This strain is viable as it also harbors a plasmid-borne copy of the 35S rRNA gene expressed from a RNAPII-dependent promoter. The wild type *RPB1* gene was then replaced with the *rpb1*-*1* allele, producing a strain that lacks RNAPI and expresses the temperature-sensitive allele of RNAPII. After shifting the *rpb1*-*1 rpa135∆* double mutant to the non-permissive temperature, the long form of *RPB2* was induced and the short form was eliminated. These results eliminate the possibility that RNAPI is required for transcription of the long form of *RPB2* mRNA when RNAPII is inactivated (Fig. 3c).

Similarly, we tested whether RNAPIII is required for eliciting the *RPB2* transcriptional response. As an initial test, we depleted RNAPIII by anchoring away the Rpc160 subunit and then induced transcriptional stress by UV treatment. As observed previously for a wild type strain, the long form of *RPB2* is preferentially produced after UV damage even when RNAPIII is depleted (Fig. 3d). As a second strategy, RNAPIII and RNAPII were simultaneously depleted by the anchor away strategy. In this case, the results are similar to those seen when RNAPII alone is depleted (compare Fig. 3e with Fig. 1b), demonstrating that RNAPIII depletion does not alter the response to transcriptional stress. These data indicate that neither RNAPI nor RNAPIII play a direct role in the transcriptional response at *RPB2*.

### Analysis of genome-wide changes in transcription after RNAPII depletion

**Fig. 4:**
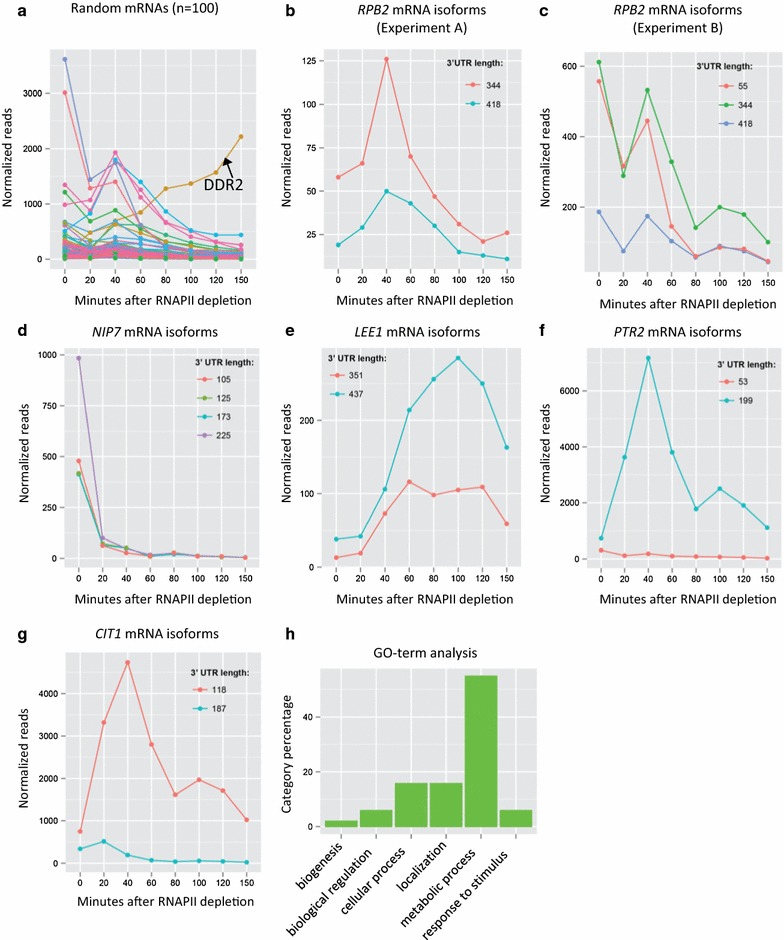
Analysis of Direct RNA Sequencing (DRS) data for mRNA isoforms after RNAPII depletion. **a** Normalized sequencing reads of 100 random transcripts in Experiment A. **b** Normalized sequencing reads of the *RPB2* mRNAs in Experiment A. **c** Normalized sequencing reads of the *RPB2* mRNAs in Experiment B. Normalized sequencing reads for the **d** *NIP7*, **e** *LEE1*, **f** *PTR2* and **g** *CIT1* genes, which represent the distinct patterns of mRNA isoform changes observed after RNAPII. **h** Gene ontology enrichment analysis of the 66 genes that were induced in both Experiment A and Experiment B after RNAPII depletion. Dataset for Experiment A and Experiment B were downloaded from NCBI (GSE52286)

**Fig. 5:**
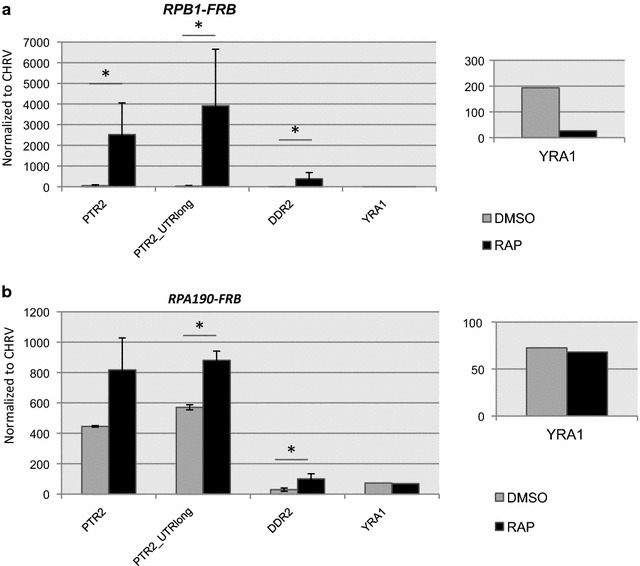
Depletion of RNAPI or RNAPII induces expression of a subset of mRNAs. RT-PCR analysis of RNA isolated from either the *RPB1*-*FRB* strain following nuclear depletion (+RAP) **a** or the *RPA190*-*FRB* strain following depletion (+RAP) **b** RNA levels were normalized to RNA from an intergenic region on chromosome V

To examine genome-wide changes in transcriptional profiles following depletion of RNAPII, we re-analyzed the datasets of Geisberg et al. [12] who used RNAPII depletion to measure the half-lives of mRNA isoforms with different polyA tails. We obtained their dataset and analyzed which mRNA species increase after depleting RNAPII. After clustering and annotating synonymous reads in each dataset, we obtained the absolute reads for all mRNA species at each time point in the two experiments (“clustered.absolute.reads.A.txt” and “clustered.absolute.reads.B.txt” in Additional file 4). Geisberg et al. [12] found 7 long-lived mRNAs with half lives >100 min (Additional file 2: Table S1). We used the median reads of these 7 mRNAs to establish the baseline of fluctuations in RNA sequencing reads and adjusted the absolute reads to this baseline (“clustered.normalized.reads.A.txt” and “clustered.normalized.reads.B.txt” in Additional file 4). Figure 4a shows the normalized sequencing reads of 100 random transcripts after RNAPII depletion in Experiment A. Most mRNAs decline after RNAPII depletion. However, unlike other transcripts, the *DDR2* mRNA with a 275 bp 3′UTR shows a continuous increase in abundance following RNAPII depletion, indicative of continuous RNA synthesis despite transcriptional inhibition. The increase in *DDR2* expression was confirmed by RT-PCR analysis of RNA isolated from cells depleted for either RNAPI or RNAPII (Fig. 5). We then looked at the dynamics of the *RPB2* mRNA isoforms in the two independent experiments (Fig. 4b, c). A long *RPB2* transcript with a 344 bp 3′UTR increases in abundance at 40 min after RNAPII depletion and then declines in both experiments. The 345th nucleotide after the *RPB2* stop codon is an adenine which is likely to be a part of the polyA tail of the *RPB2* mRNA, thus the 344 bp 3′UTR mRNA is essentially identical to the 345 bp 3′UTR mRNA identified in our previous study.

To identify additional mRNAs that are induced by RNAPII depletion, we searched for induced transcripts that were detected in both experiments A and B in the Geisburg et al. dataset [12]. We found 66 genes (70 isoforms) that were induced following RNAPII depletion (Additional file 4 “induced.normalized.common.txt”). The gene selection criteria were stringent in order to identify only those genes that are clearly induced. *RPB2* is not represented in this list due to the transient nature of its induction and the apparently high level of expression measured at time 0 in experiment B (Fig. 4b, c). Most genes have all of their mRNA isoforms repressed after RNAPII depletion (~80 % in both experiments), as represented by the *NIP7* gene (Fig. 4d). However, among the 66 genes induced by transcriptional stress, a subset of genes (10–20 % depending on the experiment) have all isoforms induced and behave similarly to the *LEE1* gene (Fig. 4e). Most other genes (80–90 %), exemplified by *PTR2*, have only one isoform preferentially induced, and show results similar to those seen for *RPB2* (Fig. 4f). Similar to the case of *DDR2*, an increase in *PTR2* mRNA levels is observed after either RNAPI or RNAPII depletion (Fig. 5). Additionally, in the case where two or more isoforms are alternatively expressed, the induced isoform is not always the long form. For example, the *CIT1* gene normally transcribes the long form and the shorter form is induced after RNAPII depletion (Fig. 4g). To characterize whether the genes induced by RNAPII depletion had any obvious functional relationship, we used PANTHER (pantherdb.org) to classify their molecular functions. As shown in Fig. 4h, most genes encode products involved in metabolic functions (Additional file 4 “induced genes.annotations.xlsx”). In addition, we also performed a search for common sequence motifs within the 5′ or 3′ UTRs, but no unique motifs were identified.

## Discussion

Recent studies indicate that transcription of protein coding genes produces a heterogeneous set of mRNAs that show alternative 3′ end formation and polyadenylation [9, 12–17]. Changes in 3′ end formation can have dramatic impacts on mRNA stability, transport, and resulting protein expression [14, 18]. A number of regulatory mechanisms have been identified that control alternative 3′ end formation, including alterations in the levels of transcription initiation or elongation [19–22]. For instance, studies in both Drosophila and budding yeast have found that slowing the elongation rate of RNAPII favors the selection of proximal termination sites, whereas enhanced elongation rates favor 3′ end formation at more distal positions [19, 21]. Such studies have led to a model whereby there is a kinetic competition between RNAPII elongation and 3′ end selection. Recently, we reported that transcription of the yeast *RPB2* gene produces mRNAs with alternative sites of polyadenylation and 3′ end formation. Furthermore, we found that a promoter-distal polyA site is preferentially used following recovery from UV damage. Here, we find that depletion of the RNAPII machinery is sufficient to induce the preferential use of the distal polyA site at the *RPB2* locus, and furthermore, a genome-wide analysis of transcript levels identifies a larger set of transcripts that are induced following RNAPII depletion. Surprisingly, we also find that depletion of RNAPI also leads to changes in 3′ end formation at *RPB2*, suggesting a more general response to transcriptional stress.

In mammalian cells, the efficiency of polyA site usage and transcription termination correlates with transcriptional activity [23]. For instance, highly expressed genes tend to utilize more proximal polyA, whereas more lowly expressed genes choose alternative, distal polyA sites. The choice of mRNA cleavage and polyA addition sites appears to be due to an increased propensity of RNAPII to pause at a proximal polyA site under conditions of high transcriptional activity [23]. Consistent with these correlative studies, we found that the depletion of RNAPII alters 3′ end usage at a subset of yeast genes. Using the anchor away system, RNAPII was rapidly removed from the nucleus, leading to efficient depletion of RNAPII from coding regions. As expected, depletion of RNAPII leads to greatly decreased densities of RNAPII at gene coding regions as well as the shutdown of a large percentage of the yeast transcriptome. However, we identified ~70 genes whose expression continues under conditions of RNAPII depletion, and many of these mRNAs also show a switch in 3′ end formation. In several cases, more distal polyA sites are used, though examples of a switch to a more proximal site was also identified.

The *RPB2* gene is an example of a gene that continues to be expressed following RNAPII depletion, and a long form of *RPB2* is induced under these conditions. This long form of *RPB2* is also induced during recovery from UV damage. Importantly, our previous study demonstrated that the long form of *RPB2* does not have an altered decay rate, indicating that continued expression is due to transcription [9]. Remarkably, RNAPII density is dramatically decreased at *RPB2* during the anchor away protocol, eliminating a simple model in which genes such as *RPB2* compete more effectively for a limiting pool of RNAPII. These lower levels of RNAPII are consistent with a model in which RNAPII density impacts alternative polyA site usage.

How can *RPB2* mRNA levels be maintained at normal or even higher levels if RNAPII levels are decreased? We entertained the possibility that other RNA polymerases might substitute for RNAPII during the depletion conditions, but co-depletion of RNAPI or RNAPIII with RNAPII did not eliminate the induction of the long form of *RPB2*. These results indicate that the residual levels of RNAPII are responsible for the continued expression of the long form of *RPB2*. This raises the possibility that these bound RNAPII molecules are engaged in a more productive mode of transcription. Interestingly, many genes that encode subunits of RNAPII are increased in expression after inactivation of the RNA exosome [24]. Furthermore, we previously reported that yeast genes with a lower density of RNAPII are not as likely to be targeted by the RNA exosome, consistent with the idea that the transcription of some genes during RNAPII depletion may be more productive [24]. Interestingly, genotoxic stress has been shown to inhibit the nonsense mediate decay pathway [25]. One possibility is that polymerase depletion may also lead to a similar type of stress that down regulates decay of a subset of transcripts. Since the induced, long *RPB2* isoform does not appear to have an altered half-life [9], such a decay mechanism may operate co-transcriptionally.

Laferte and colleagues [26] reported that increased levels of RNAPI activity not only induce expression of the 35S rRNA but also the RNAPII-dependent expression of ribosomal protein genes. They suggested the possibility of communication between RNAPI and RNAPII that would ensure coordinated expression levels of ribosomal components. Remarkably, we find that depletion of RNAPI leads to a RNAPII-dependent induction of the *RPB2* gene and a change in the selection of the 3′ end. This response occurs rapidly (<60’), so it likely occurs prior to any decreases in translational capacity. We also find that the *PTR2* and *DDR2* transcripts are also increased following either RNAPI or RNAPII depletion, though the magnitude of the transcriptional induction is much larger when RNAPII is depleted. We favor a model proposed by Lafrete [26] and colleagues in which transcription of 35S rRNA or the structure of the nucleolus is monitored in some way by the RNAPII machinery leading to an altered transcriptional activity.

## Conclusions

We demonstrate that depletion of RNAPII and RNAPI, but not RNAPIII results in a transcriptional stress response that triggers the induction of specific mRNAs and that many of the mRNAs induced also exhibit a change in the polyadenylation site that is preferentially used. Thus, transcriptional stress response must result in modification of the transcriptional machinery to cause these changes.

## Methods

### Yeast strains and plasmids

Yeast strains and plasmids used in this study are listed in Table 1. Strain construction details are described below. Primer sequences are listed in Additional file 3: Table S2.

**Table 1:** Yeast strains used in this study

Strain	Original name, genotype (annotation)	Notes	References
MVY150	W303-1B, MATα *ade2*-*1 trp1*-*1 can1*-*100 leu2*-*3, 112 his3*-*11,15 ura3*-*1*		[7]
MVY451	SYY9, MATa *ade2*-*1 his3*-*11, 15 leu2*-*3, 112 trp1*-*1 ura3*-*1 can1*-*100 rpb1*-*1* (derived from W303)		[27]
MVY845	NOY408-1a, MATα *rpa135::LEU2 ade2*-*1 ura3*-*1 trp1*-*1 leu2*-*3,112 his3*-*11 can1*-*100* carrying pNOY102 (*GAL7*-35S rDNA *URA3*)		[28]
MVY851	MATα rpa135::LEU2 *ade2*-*1 ura3*-*1 trp1*-*1 leu2*-*3,112 his3*-*11 can1*-*100* carrying pNOY102 (*GAL7*-35S rDNA *URA3*) *rpb1*-*1*		This study
MVY858	HHYy168, MATα *tor1*-*1 fpr1::NAT RPL13A*-2x*FKBP12::TRP1*		[11]
MVY859	HHY170, MATα *tor1*-*1 fpr1::NAT RPL13A*-2x*FKBP12::TRP1 RPO21*-*FRB::KANMX6*		[11]
MVY860	MVY858 with *RPA190*-*FRB::KANMX6*		This study
MVY862	MVY858 with *RPC160*-*FRB::KANMX6*		This study
MVY868	MVY858 with *TBP1*-*FRB::KANMX6*		This study
MVY872	MVY859 with *RPA190*-*FRB*-*HIS3MX*		This study
MVY874	MVY859 with *RPC160*-*FRB*-*HIS3MX*		This study
MVY879	MVY872 with *RPC160*-*FRB*-*LEU2*		This study
CY1940	MVY858 with *RPA190* FLAG	Pol I AA	This study
CY2032	MVY859 with *RPA190* FLAG	Pol I AA Pol II AA	This study

To construct strain MVY851 (*rpa135∆ rpb1*-*1*), we mated MVY845 and MVY451, sporulated the diploid cells, and dissected the tetrads onto YEP-galactose. Spore colonies that grew on Leucine-dropout Galactose plates (*rpa135::LEU2*) and could not grow at 37 °C (*rpb1*-*1*), were further tested as follows. To test *rpa135::LEU2*, we PCR amplified the genomic DNA with primer RPA135-f (TCAAACTTACTTCAGCTGTCTCG) and primer RPA135-r (GGCTTCCAAACCCTTTAGGT), digested the PCR fragment with restriction enzyme XbaI and separated the digested DNA on agarose gels. The *RPA135* wild type strain had 3 bands of 1957 bp, 1101 bp, and 886 bp, the *rpa135::LEU2* mutant clone had a 3.7 kb band. To confirm the *rpb1*-*1* allele, we PCR amplified the genomic DNA with RPB1f (GTTGATCATCCGTTGTCGTG) and RPB1-4955r (GGTGACGTTGGGCTGTAACT), and sequenced with primer RPB1r (CATTTGAACCCGAATCAACC). A G≥A point mutation at bp 4310 of the *RPB1* gene was confirmed in the temperature sensitive clone.

To construct strain MVY860 (*RPA190*-*FRB::KANMX6*), we PCR amplified the *KanMX6* gene from pFA6a-FRB-KanMX6 using primers RPA190aaf (GAACAATGTTGGTACGGGTTCATTTGATGTGTTAGCAAAGGTTCCAAATGCGGCTcggatccccgggttaattaa) and RPA190aar (CTCCTTCAAATAAACTAATATTAAATCGTAATAATTATGGGACCTTTTGCCTGCTTgaattcgagctcgtttaaac) to produce DNA fragment R1, PCR amplified a 403 bp fragment from the *RPA190* gene using primers RPA190-5586f (TGTGGGATCAAGAGGCATTT), RPA190-5589r(CGCATTTGGAACCTTTGCTA) to produce DNA fragment A1, PCR amplified a 394 bp fragment from the *RPA190* gene using primers RPA190-5999f (CAGGCAAAAGGTCCCATAAT) and RPA190-6393r (ATTGGCCGTTCCTTCAAATA) to produce DNA fragment A2, then assembled purified DNA fragments A1, A2 and R1 using overlap extension PCR (one cycle of at 95 °C for 2 min, then 13 cycles at 95 °C for 20 s, 55 °C 20 s, and 72 °C 30 s, followed by one cycle at 72 °C for 4 min) to produce the DNA molecule F1. F1 was then used as a template in which we PCR amplified an 2870 bp DNA using primers RPA190-5600-SalI (GCGC-GTCGAC-GCATTTATCGACGTTGATGGT) and RPA190-6309r-SacII (GGCA-CCGCGG- CCAGCACGTAATCGAGAAAA). The resultant 2870 bp PCR product was then digested the with SalI and SacII and inserted between the SalI and SacII sites of the pBluescript SK vector plasmid to produce pMV1374. pMV1374 was then digested with SacII, SalI, and DrdI to release the 2768 bp fragment containing sequences homolgous to sequences flanking *RPA190* but containing the *FRB*-*KANMX6* cassette. The gel purified 2768 bp fragment was then transformed into strain MVY858 and selected for clones that are resistant to G418 and sensitive to Rapamycin. The resulting clones were confirmed by PCR amplifying the genomic DNA using primers FRB-r (CCTTCATGCCACATCTCATG) and RPA190-5528f (CCAGAACCTGAAAACGGAAA) and looking for amplification of the signature 564 bp PCR product.

To construct strain MVY862 (*RPC160*-*FRB::KANMX6*), we PCR amplified the *KanMX6* gene from pFA6a-FRB-KanMX6 using primers RPC160aaf (TCCTAAGCGATGTCTATTTGAAAGTCTCTCAAATGAGGCAGCTTTAAAAGCGAACcggatccccgggttaattaa) and RPC160aar (TGACTGTGGTAGAAAAATAATACAAATGCTATAAAAAAGTTTAAAAACGACTACTgaattcgagctcgtttaaac), transformed the strain MVY858 with the purified PCR fragment, and selected the transformants on YPD plates containing 200ug/ml G418, and confirmed the RPC160-FRB::KANMX6 construct by PCR amplifying the characteristic 806 bp fragment using primers KanMX-905 (TTTGATGACGAGCGTAATGG) and RPC160 + 233r (TCAGCTTGTGAGTGCATACCA).

To construct strain MVY872 (*RPB1*-*FRB::KANMX6 RPA190*-*FRB*-*HIS3MX)*, we first replaced the KanMX6 marker in pMV1374 with the *HIS3MX6* marker with the following steps: digestion of pMV1374 with BamHI and EcoRI and purification of the 3.3 kb vector, digestion of the pFA6a-*FRB*-*HIS3MX6* with BamHI and EcoRI to release the 1.9 kb insert containing the HIS3MX6 gene. The vector and insert were then ligated to obtain pMV1377 (pBluescriptSK with *RPA190*-*FRB*-*HIS3MX6*). This plasmid was then sequenced using T7/T3 primers to confirm its structure. Then we digested pMV1377 with SacII and SalI and DrdI to release the 2655 bp *RPA190*-*FRB*-*HIS3MX6* fragment and used the fragment to transform MVY859 and selecting on the Histidine-dropout minimum plate. Clones were confirmed by PCR amplification with primers FRBr (CCTTCATGCCACATCTCATG) and RPA190-5528f (CCAGAACCTGAAAACGGAAA) with expected amplicon of size 600 bp.

To construct strain MVY874 (*RPB1*-*FRB::KANMX6 RPC160*-*FRB*-*HIS3MX*), we first cloned the *RPC160*-*FRB::KANMX6* construct from MVY862 into pBluscriptSK to make plasmid pMV1379 with the following steps, PCR amplification was performed using the genomic DNA of strain MVY862 as template and primers RPC160-4859-SalIf (ACGC-GTCGAC-GATGACGGCAAGAGGGAAT) and RPC160-5866-SacIIr (GGCA-CCGCGG-TTGCCTCTCAATGCCCATA). The resultant amplicon was then ligated into the pBluescriptSK vector with SalI and SacII to construct plasmid pMV1379. It structure was confirmed by DNA seuquencing using T7/T3 primers. Plasmids pMV1379 and pFA6a-FRB-HIS3MX6 were then digested with BamHI and PmeI and ligated to swap the HIS3MX6 and KanMX6 markers to make pMV1382 (RPC160-FRB-HIS3MX6) sequencing using T7/T3 primers confirmed its structure. pMV1382 was then digested with SacII, SalI, and DrdI to release the 2953 bp fragment, containing RPC160 homologous sequences flanking the FRB-HIS3MX6 cassette, which was used to transform strain MVY859 and selecting on Histidine dropout plate for His + clones. The presence of the *RPC160*-*FRB*-*HIS3MX* construct was confirmed by amplifying the signature 700 bp fragment using primers FRBr (CCTTCATGCCACATCTCATG) and RPC160-4796f (GCGCTTCCTGATGTTGTTGT).

To construct strain MVY879 (MVY872 with *RPC160*-*FRB*-*LEU2*), we first replaced the KanMX6 marker in pMV1379 with the *LEU2* gene from pRS315 using the following step: PCR amplification of the *LEU2* gene from plasmid pRS315 using primers LEU2-BglIIf (GCGC-AGATCT-ACCCTCGAGGAGAACTTCTAGTATATC) and LEU2-PmeIr (GTAC-GTTTAAAC-TCGACTACGTCGTAAGGCC). The amplicon was digested with BglII and PmeI and inserted into the vector pMV1379 between the cloning sites BglII and PmeI to make the plasmid pMV1381. Its structure was confirmed by sequencing using primers T7/T3. Plasmid pMV1381 was then digested with SalI and SacII to release the *RPC160*-*FRB*-*LEU2* fragment (3872 bp), which was used to transform the yeast strain MVY872. Transformants were selected on Leucine-dropout plates and positive clones are confirmed by PCR amplifying the signature 4 kbp fragment using primers RPC160+233r (TCAGCTTGTGAGTGCATACCA) and RPC160-4796f (GCGCTTCCTGATGTTGTTGT).

### Anchor-away and UV irradiation

Yeast cells were cultured in YPD with 200 µg/ml of G418 overnight, centrifuged and diluted in fresh YPD to OD = 0.1 and cultured for another 6 h. If cells were to be UV irradiated, cells were collected and resuspended in PBS and irradiated with UV at 1.71 J/m^2^/s for 42 s (70 J/m^2^), then resuspended in YPD and cultured at 30 °C in the dark. Rapamycin was added to the culture to a final concentration of 8 µg/ml to induce anchor-away.

### Heat shock at 37 °C

Heat shock induction was performed by culturing cells at 23 °C, then mixing the cells with an equal volume of YPD at 51 °C to immediately raise the incubation temperature to 37 °C. The cultures were then maintained at 37 °C.

### Northern blot analysis

Procedures of yeast RNA extraction (hot phenol method) and Northern analysis are as described previously [29]. Northern blot images were acquired on a BAS-2500 Image Scanner (Fujifilm) and processed using Multi Gauge 3.0 (Fujifilm)

### RT-PCR analyses of RNA

Yeast were grown in yeast extract peptone (YEP) media with 2 % glucose at 30 °C at an OD600 of 0.8, ensuring equal cell number and cell pellets were snap-frozen in liquid nitrogen until RNA extraction. All samples in one biological replicate were processed together for RNA extraction to minimize variations across mutants and a minimum of two biological replicates were performed for each sample. The cell pellets were thawed on ice and 200 µL of lysis buffer (50 mM Tris pH7-7.4, 130 mM NaCl, 5 mM EDTA, 5 % SDS), 200 µL of Phenol (pH 4.0): Chloroform: Isoamylalcohol (PCI, 25:24:1) and 200 µl of glass beads (by volume) were added before vortexing in the cold room for 20 min at maximum speed. Samples were spun for 15 min at 4 °C at >13,000 rpm to separate the organic and aqueous phases. The upper aqueous phase was transferred to a fresh precooled 1.7 ml tube; an equal amount of PCI was added before shaking the tubes vigorously, followed by centrifugation at maximum speed. This step was repeated once more and then the aqueous layer was mixed with an equal amount of Chloroform: Isoamyl alcohol (CI, 24:1) to remove any residual phenol. The tubes were shaken and spun as above. Phase Lock Heavy tubes (5 Prime) were prepared with a short pre-spin (30 s, 3000 rpm) and the upper aqueous layer from the RNA samples added along with an equal quantity CI. The phases were mixed by inverting the tubes vigorously, taking care to avoid any vortexing, and spun at >13,000 rpm for 2 min. The upper layer was transferred to fresh precooled 1.7 ml tubes containing 1/20th the volume of 3 M sodium acetate (pH 4.2), 2 volumes of 100 % ethanol and inverted. RNA was precipitated at −20 °C for 30 min, spun at 15 min at >13000 rpm and supernatant discarded. The pellet was rinsed with 80 % ethanol, spun for 2 min and the supernatant discarded. The pellets were allowed to air dry by inverting for ~30 min, resuspended in 50–100 μl of DEPC water and quantified using a Nanodrop. Ideally, A260/280 for RNA ~2 and A260/230 ~2, and it is important to note that lower ratios might indicate organic contamination. Integrity of the RNA was confirmed on a 2 % Agarose gel that showed a light smear with 2 bands for the high molecular weight ribosomal subunits and 3 bands for the low molecular weight RNAs. Total RNA was treated for at least 1 h with TURBO DNase (TURBO DNA free kit, Ambion #1907) by incubation for 30 min in the 37 °C water bath in 50 µL reactions with ≤25 µg of RNA in each tube. Care was taken to avoid sample agitation as DNase is extremely heat labile. 10X Inactivation reagent was added after the incubation and frequently tapped to ensure uniform mixing. Samples were centrifuged for 2 min at >13,000 rpm and the supernatant was used for the RT-PCR. Total RNA of 60 ng per well was determined to amplify signal in the linear range for most sample/primer sets. Primers were designed to be specific using Primer3 qRT-PCR settings and melt-curve analysis was performed in each run to confirm unique product amplification. The One-step RT-PCR reaction mix contained 2X Sybr Green PCR mix from Invitrogen (SYBR Green, ROX, dNTPs), Superscript RT III, primers). In addition to using equal RNA amounts, signal from all primer sets were normalized to a non-transcribed region on CHRV.

### Analysis of the DRS dataset

Direct RNA sequencing (DRS) dataset for Experiment A and Experiment B were downloaded from NCBI (GSE52286). Reference yeast genome sequence file (sacCer3, 2008 June assembly) was downloaded from yeastgenome.org. Yeast gene annotations (sgdGene2008.txt and sgdToName2008.txt) were downloaded from UCSC Table browser. The sequence analysis was done using custom-written R programs, which are available upon request. For transcripts that were undetectable at only some of the 8 time points, we set the read counts as 1 to avoid missing values. Nearby sequencing reads (distance less than 15 base pairs) were clustered to the most abundant isoforms. We set the minimum total reads of the clustered absolute transcripts in all 8 experiments to 300 reads and set the maximum 3′UTR length as 1000 bp. Induced transcripts were defined as transcripts whose normalized reads following Pol II depletion were at least twofold higher than at time 0 for at least 4 time points in the 7 time series in both Experiment A and Experiment B. To search for sequence motifs, Multiple Em for Motif Elicitation (MEME; http://meme-suite.org/tools/meme) was used to probe an 800 bp 5′UTR from the 70 induced gene isoforms as well as 70 random genes. MEME was also used to probe sequences from the protein stop codon to the polyA site.

### ChIP experiments

Yeast strains were grown in rich media with 2 % glucose at 30 °C and either DMSO or Rapamycin (8 μg/ml final concentration) was added for 60 min before fixation with 1.2 % formaldehyde. Cells were quenched with 2.5 M glycine, centrifuged, rinsed with cold water and stored at −80 °C until chromatin preparation. Chromatin preparation, immunoprecipitation and DNA extraction were performed as described in [30]. The anti-CTD antibody (1 μL for 100μL chromatin) was used to immunoprecipitate Pol II. The anti-Rpc34 antibody (1 μL for 100μL chromatin) was used to immunoprecipitate Pol III. The Pol I subunit *RPA190* was C-terminally tagged with a FLAG tag and a anti-FLAG antibody used for immunoprecipitaion.

## Additional files

10.1186/s12867-016-0074-8 Confirmation of RPB2 Northern blot data by qRT-PCR. The schematic in panel A indicates locations of the primers that amplify the long RPB2 mRNA (purple; amplifies only the 4297 nt long form) and the short RPB2 mRNA (green; amplifies both 4010 nt and 4297 nt forms). Panel B presents the RT- PCR analysis of RNA isolated from the RPB1-FRB strain following nuclear depletion (+RAP). RNA levels were normalized to RNA from an intergenic region on chromosome V as done in Figure 5.
10.1186/s12867-016-0074-8 mRNAs with long half-lives from Geisberg et al. [12]. The seven long lived mRNA species identified by Geisberg et al. that were used in this study to establish the baseline and the relevant features of each mRNA are listed in Supplementary Table.
10.1186/s12867-016-0074-8 Primer sequences. Sequences of all primers used in this study and the experiment in which they were used are listed in Supplementary Table S2.
10.1186/s12867-016-0074-8 Gene expression data files. Text files of all absolute and normalized reads, as described in the text, are contained within the gene expression data files.

## Abbreviations

RNAPI: RNA polymerase I
RNAPII: RNA polymerase II
RNAPIII: RNA polymerase III
TCR: transcription coupled repair
polyA: polyadenylation
*Tbp1*: TATA binding protein 1
ChIP: chromatin immunoprecipitation
YEP: yeast extract peptone
DRS: direct RNA sequencing
