# Supplementary material for: RNA polymerase II depletion promotes transcription of alternative mRNA species
Source: BMC Mol Biol. 2016 Aug 30;17(1):20. doi: 10.1186/s12867-016-0074-8 (PMC5004267; doi:10.1186/s12867-016-0074-8)
Supplement: Supplementary file 1 — 10.1186/s12867-016-0074-8 Confirmation of RPB2 Northern blot data by qRT-PCR. The schematic in panel A indicates locations of the primers that amplify the long RPB2 mRNA (purple; amplifies only the 4297 nt long form) and the short RPB2 mRNA (green; amplifies both 4010 nt and 4297 nt forms). Panel B presents the RT- PCR analysis of RNA isolated from the RPB1-FRB strain following nuclear depletion (+RAP). RNA levels were normalized to RNA from an intergenic region on chromosome V as done in Figure 5. [file 12867_2016_74_MOESM1_ESM.pptx]

## Slide 1
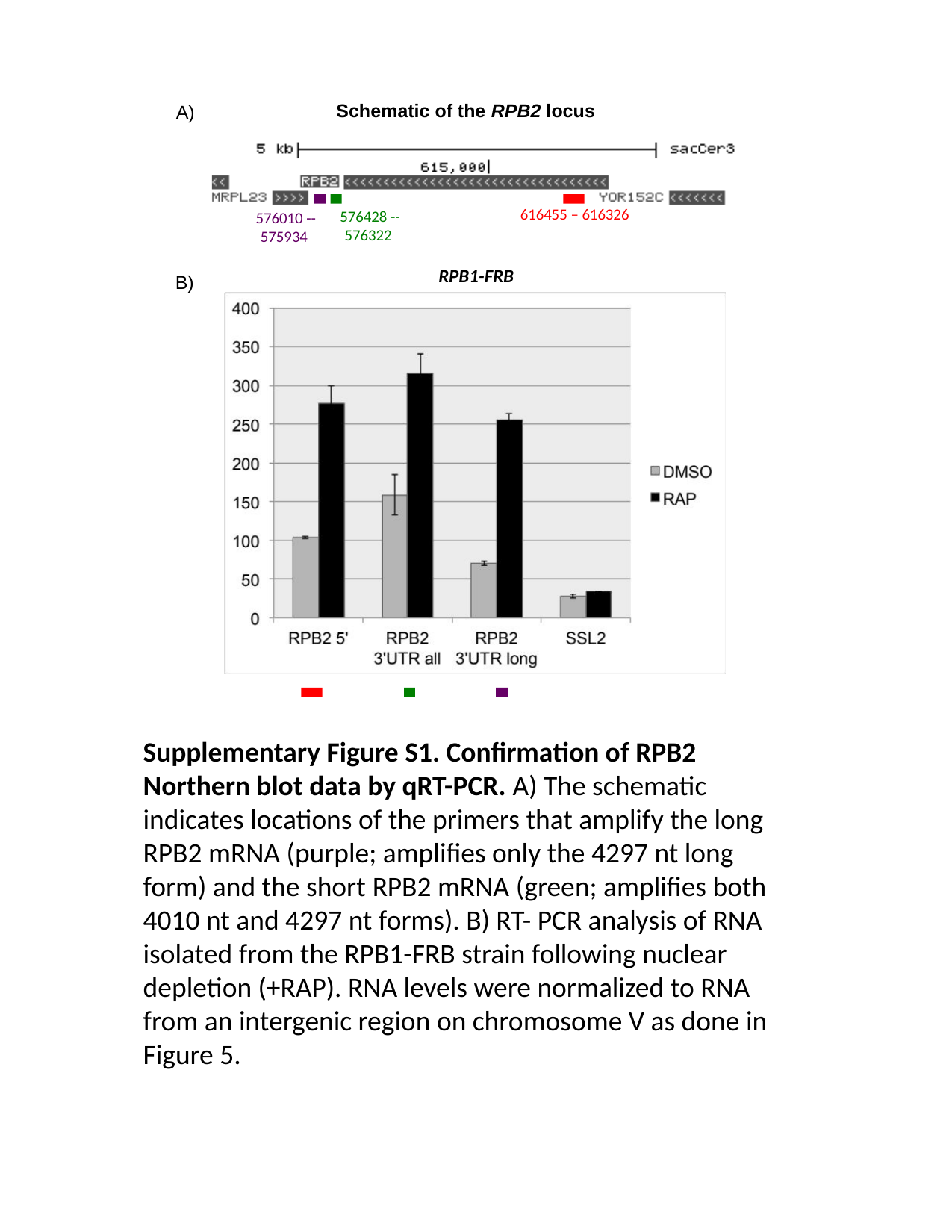

Schematic of the RPB2 locus
A)
576010 -- 575934
616455 – 616326
576428 -- 576322
RPB1-FRB
B)
Supplementary Figure S1. Confirmation of RPB2 Northern blot data by qRT-PCR. A) The schematic indicates locations of the primers that amplify the long RPB2 mRNA (purple; amplifies only the 4297 nt long form) and the short RPB2 mRNA (green; amplifies both 4010 nt and 4297 nt forms). B) RT- PCR analysis of RNA isolated from the RPB1-FRB strain following nuclear depletion (+RAP). RNA levels were normalized to RNA from an intergenic region on chromosome V as done in Figure 5.
