# Supplementary material for: RNA polymerase II depletion promotes transcription of alternative mRNA species
Source: BMC Mol Biol. 2016 Aug 30;17(1):20. doi: 10.1186/s12867-016-0074-8 (PMC5004267; doi:10.1186/s12867-016-0074-8)
Supplement: Supplementary file 2 — 10.1186/s12867-016-0074-8 mRNAs with long half-lives from Geisberg et al. [12]. The seven long lived mRNA species identified by Geisberg et al. that were used in this study to establish the baseline and the relevant features of each mRNA are listed in Supplementary Table. [file 12867_2016_74_MOESM2_ESM.docx]

**Supplementary Table S1: mRNAs with long half-lives from** **Geisberg et al**

| Standard Name | Chromosome | Strand | cdsStart | cdsEnd | 3'UTR lengths | Half life (min) |
| --- | --- | --- | --- | --- | --- | --- |
| FUS1 | 3 | + | 71802 | 73341 | 90 | 109 |
| YDR262W | 4 | + | 993130 | 993949 | 196 | 105 |
| SNA2 | 4 | + | 1490588 | 1490828 | 222 | 107 |
| YFR052C-A | 6 | - | 253415 | 253721 | 4 | 108 |
| YGR146C | 7 | - | 784227 | 784863 | 231 | 131 |
| SSL2 | 9 | - | 80509 | 83041 | 250 | 112 |
| CPA1 | 15 | + | 882896 | 884132 | 64 | 127 |
