# Supplementary material for: RNA polymerase II depletion promotes transcription of alternative mRNA species
Source: BMC Mol Biol. 2016 Aug 30;17(1):20. doi: 10.1186/s12867-016-0074-8 (PMC5004267; doi:10.1186/s12867-016-0074-8)
Supplement: Supplementary file 3 — 10.1186/s12867-016-0074-8 Primer sequences. Sequences of all primers used in this study and the experiment in which they were used are listed in Supplementary Table S2. [file 12867_2016_74_MOESM3_ESM.docx]

**Suppementary Table S2. Primer sequences**

| **Primer name** | **Sequence** | **Figure** |
| --- | --- | --- |
| MR257_RPB2_5'_F | CAGTTGGCTCAACATACTACCG | Figure_2_ChIP |
| MR258_RPB2_5'_R | CTTGTGGATACAACGCATGG | Figure_2_ChIP |
| MR259_RPB2_3'_F | GTATTTGCGGGCTGATGAC | Figure_2_ChIP |
| MR260_RPB2_3'_R | AATAACTTCGCGGCGTATG | Figure_2_ChIP |
| MR237_*tRNA^Phe^(GAA)P2^§^* | GACGCTTGGACCATTTATAAAGCAC | Figure_2_ChIP |
| MR238_*tRNA^Phe^(GAA)P2^§^* | CCATAAGAGAAGGAGCAGTCAAGTTCA | Figure_2_ChIP |
| ACT1 5'_F | GGCCAAATCGATTCTCAAAA | Figure_2_ChIP_Figure_5_RNA |
| ACT1 3'_R | GCCTTCTACGTTTCCATCCA | Figure_2_ChIP_Figure_5_RNA |
| HMR-Ya-F (1) | CATCAATATCACCCCAAGCAC | Figure_2_ChIP |
| HMR-Ya-R (1) | CCTTGGAATTAAGGCTTTGC | Figure_2_ChIP |
| RDN37-F (5’end-1) | TAACGATACAGGGCCCATTC | Figure_2_ChIP |
| RDN37-R (5’end-1) | GACTTGCCCTCCAATTGTTC | Figure_2_ChIP |
| DDR2_F | TTTCTGCCATCTCTGTCTTCG | Figure_5_RNA |
| DDR2_R | TTACTCGTGGTGTTGGATGC | Figure_5_RNA |
| PTR2_UTRlong_F | ACGTTGACGGGAGTTTTCTG | Figure_5_RNA |
| PTR2_UTRlong_R | TCGATAGCAATGGAAGAGGAG | Figure_5_RNA |
| PTR2_2_F | TGTTCTGGTTGTGCTTCAGG | Figure_5_RNA |
| PTR2_2_R | AGCTTTAGGTGCGGAAATTG | Figure_5_RNA |
| YRA1_F_MR477 | AACGAAAGGGGCCAATCTAC | Figure_5_RNA |
| YRA1_R_MR478 | TCTTTCAACAGCCCTTCTGG | Figure_5_RNA |
| SUB1_F_MR479 | TGGAGCCACGTTCAAAGAAG | Figure_5_RNA |
| SUB1_R_MR480 | GCGTTCCTCTTGTTGTTTCC | Figure_5_RNA |
| ChrV 12848 MR288 | CAAGTTTGGTGAGATAGTTTACGC | Figure_5_RNA |
| ChrV 13002 MR289 | CTGGCGTGCGCATATAAGACTG | Figure_5_RNA |
